# Wound Healing Activity of *Carallia brachiata* Bark

**DOI:** 10.4103/0250-474X.58184

**Published:** 2009

**Authors:** B. Krishnaveni, V. Neeharika, S. Venkatesh, R. Padmavathy, B. Madhava Reddy

**Affiliations:** Department of Pharmacognosy, G. Pulla Reddy College of Pharmacy, Mehdipatnam, Hyderabad-500 028, India

**Keywords:** *Carallia brachiata*, *Rhizophoraceae*, stem bark, wound healing activity

## Abstract

The stem bark of *Carallia brachiata* was studied for wound healing activity. The bark was extracted with petroleum ether, ethyl acetate and methanol successively. All the extracts were screened for wound healing activity by excision and incision models in Wistar rats. The ethyl acetate and methanol extracts were found to possess significant wound healing activity. The extracts revealed the presence of sterols or triterpenoids, flavonoids, phenols, tannins, carbohydrates, fixed oils and fats.

*Carallia brachiata* Merrill (Rhizophoracea) is a large tree occurs through out India up to an altitude of 1300 m and often planted as ornamental tree[1]. The fruits and seeds are edible. The seeds contain oil which is used as a substitute for *ghee* in Karnataka. An infusion of leaves is taken like tea and together with a mixture of benzion, turmeric and rice dust is used in treatment of sapraemia[2]. The bark is mentioned to be useful in the treatment of itching, cuts and wounds, oral ulcers, inflammation of the throat and stomatitis[1,3]. From the bark, new proanthocyanidins named carallidin, mahuanin and parahydroxy benzoic acid were isolated. Proanthocyanidins were reported to possess free radical scavenging activity[4]. A new megastigmane diglycoside (3-hydroxy-5,6-epoxy-β-ionol-3-O-β-apiofuranosyl(1→6)-β-glucopyranoside), two megastigmanes, condensed tannins, flavanoids and glyceroglycolipids were isolated from the leaves[5]. The alkaloid hygroline was also reported from the leaves[5]. In the present study the bark is evaluated for wound healing activity to prove scientifically its traditional use in the treatment of cuts and wounds.

The bark was collected from Tirupati forest, AP, India in September 2006 and authenticated at the Department of Botany, Sri Venkateshwara University, Tirupati, India. A voucher specimen (CB-10-06) has been deposited in the Pharmacognosy and Phytochemistry laboratory of G. Pulla Reddy College of Pharmacy, Hyderabad, India.

The powdered bark (500 g) was extracted successively with petroleum ether (yield 0.62% w/w), ethyl acetate (yield 3.34% w/w) and methanol (yield 6.2% w/w) by cold maceration at room temperature (30±2°). The extracts were tested for various phytoconstituents as per the reported procedures[6,7]. Five percent petroleum ether, ethyl acetate and methanol extract ointments were prepared by using combination of PEG 400 and 6000 as ointment bases. Screening for wound healing activity was performed by excision and incision wound models in Wistar rats. The experimental protocol was approved by Institutional Animal Ethics Committee (IAEC) of G. Pulla Reddy College of Pharmacy (Reg No. 320/CPCSEA).

Wistar rats of either sex weighing 150-200 g were inflicted with excision wounds as described by Morton and Malone[8]. Circular wounds of 50 mm^2^ were inflicted on the depilated dorsal thoracic region under mild ether anesthesia. The areas (mm^2^) of the wounds were measured by placing a transparent polythene graph paper over the wound. This was taken as the initial wound area reading. The wound was left undressed, open to environment. The animals were divided into 5 groups of 6 animals each. The group I served as the control and received ointment base, group II served as reference standard and received nitrofurazone ointment (0.2% w/w), group III, IV and V were treated with petroleum ether, ethyl acetate and methanol extracts (5% w/w in PEG 400:6000) ointments, respectively. The ointments were applied topically on the wounds once in a day. The parameters studied were percentage closure of excision wound and time of epithelization. The wound area was measured on 2^nd^, 4^th^, 6^th^, 8^th^, 10^th^ and 12^th^ day until healing was complete. The percentage of wound closure was calculated with respect to the initial wound area. The period of epithelization was calculated as the number of days required for complete closure of wound.

In the Incision wound model two para vertebral straight incisions of 6 cm each were made on the depilated skin on either side of vertebral column of the rat as described by Ehrlich and Hunt[9]. Care was taken to see that the incisions were at least 1cm lateral to the vertebral column. After complete haemostasis, the wounds were closed by means of interrupted sutures of 1 cm apart. The grouping and treatment of animals is similar to that of excision wound model. Sutures were removed on 8^th^ post wounding day and tensile strength was determined on 10^th^ post wounding day according to the method of Lee[10]. All the values are expressed as mean±SEM and subjected to student “t” test.

The preliminary phytochemical screening showed presence of steroids, terpenoids, tannins and carbohydrates in ethyl acetate and methanol extracts, while fixed oils and fats were present only in petroleum ether extract. In the excision wound healing model, the ethyl acetate and methanol extracts have shown considerable wound contraction from fourth day onwards. The wound healing is progressive and almost complete on 12^th^ day. The ethyl acetate extract has shown comparable wound contraction (98%) to that of standard drug nitrofurazone (95%). The methanol extract has shown slightly less contraction (89%) than ethyl acetate extract. However the petroleum ether extract activity is very less and not consistent. Initially it has shown contraction (19%) up to 4^th^ day and later there is no further progress in the wound contraction. The detailed results are presented in Table 1. In the incision wound model also the results are in the same pattern as that of excision model. The ethyl acetate extract has shown maximum tensile strength (762.53 g) on the 10^th^ day which is comparable to that of the standard drug nitrofurazone (740.69 g) followed by methanol extract (514.27 g). However there is no difference in the tensile strength of wound of the control group animals and petroleum ether extract treated animals. The results are presented in Table 2.

**TABLE 1:** EFFECT OF *CARALLIA BRACHIATA* BARK EXTRACTS ON EXCISON WOUND IN RATS

Post wounding days	Wound area (mm^2^)				

	Group I Control	Group II Nitrofurazone	Group III Pet. ether ext.	Group IV Ethyl acetate Ext.	Group V Methanol ext.
0	50.24±1.12	50.24±1.32	50.84±1.82	63.58±1.50	50.24±0.89
2	50.10±1.30	41.20±41* (17)	49.82±1.67 (2)	59.36±1.64* (6)	50.20±0.90
4	40.20±0.89 (19)	33.16±0.96* (33)	41.18±2.40* (19)	56.71±0.76* (11)	42.20±0.72* (16)
6	38.25±1.81 (23)	22.46±1.13* (55)	39.65±1.60* (22)	24.21±0.98* (61)	35.46±0.71* (29)
8	38.25±1.91 (23)	15.89±1.82* (68)	39.65±1.80* (22)	9.61±1.54* (84)	16.71±2.10* (66)
10	37.22±2.17 (25)	9.48±1.93* (81)	39.14±1.20* (23)	5.54±2.70* (91)	11.69±2.71* (76)
12	36.45±3.20 (27)	2.52±2.76* (95)	38.13±0.89* (25)	1.22±2.80* (98)	5.53±1.35* (89)

Values are mean ± SEM of 6 animals in each group.

* *P*< 0.05 compared with initial wound area (0 day) in the respective group. Figures in parenthesis indicate the percentage of wound contraction.

**TABLE 2:** EFFECT OF *CARALLIA BRACHIATA* BARK EXTRACTS ON INCISION WOUND IN RATS

Group	Treatment	Tensile strength (g)
I	Ointment base (Control)	333±8.48
II	Nitrofurazone Ointment (standard 0.2% w/w)	740±7.82*
III	Petroleum ether extract Ointment (5% w/w)	335±8.9
IV	Ethyl acetate extract Ointment (5% w/w)	762±6.14*
V	Methanol extract Ointment (5% w/w)	514±7.46*

Values are mean±SEM of 6 animals in each group.

* P< 0.05 with respective to control group

In both the models the ethyl acetate and methanol extracts were found to possess good wound healing activity and the activity is comparable to standard drug nitrofurazone at the tested dose level of 5% extract ointments. The higher tensile strength evidenced in the incision wound model indicates that the bark extracts caused enhanced collagen maturation. Collagen is the major protein of the extra cellular matrix which gives strength to the healing wound[11]. The healing of wound is also associated with many other processes such as coagulation, cellular proliferation, anti-inflammatory and tissue remodeling. The activity of the bark shown in the incision model could be through any of these processes. As the chemical tests reveal the presence of many phytoconstituents, further work is needed to establish the active constituents. The present study provides a scientific base and validates ethnomedical use of *Carallia brachiata* bark in the treatment of sapraemia.
